# New Appliances and Things Medical

**Published:** 1895-02-16

**Authors:** 


### NEW APPLIANCES AND THINGS MEDICAL.

[We shall be glad to receive, at our Office, 428, Strand, London, W.C., from the manufacturers, specimens of all new preparations and appliances which may be brought out from time to time.]

### AUSTRALIAN BRANDY.

(Joshua Brothers, 26, Mark Lane, E.C.)

This new brandy is certainly deserving of attention, inasmuch as a considerable quantity of the so-called brandy introduced into this country is not made from the grape, but consists of a spirit obtained by distillation from the fermented liquors made from grains and potatoes. Australian Brandy (Joshua Brothers) comes from the colony of Victoria, and is exported under the supervision of the Department of Trade and Customs and is accompanied by a certificate from the Excise Office stating that it is a pure grape brandy. We understand that the brandy is imported in bulk to this country where it is bottled and diluted to the strength which it is customary to use. From our analysis we find that the alcoholic strength is well above that required by the authorities, and that it leaves a residue of about one per cent., consisting of sugar and colouring matter. On burning the residue gave the characteristic odour of a French brandy and yielded an ash which was less than 0.01 per cent. Many of the brandies of commerce have been found to contain minute quantities of copper, but this sample was practically free from this objectionable constituent. Owing to the serious collapse of the French brandy industry through the ravages of the phylloxera, there is room in this country for a genuine brandy, and this Australian product seems to fulfil all the requirements and for medical purposes leaves nothing to be desired.

### SPINAL SUPPORT.

Mr. C. G. Gumpel, of 70, Newman-street, W., has favoured us with a small pamphlet giving a full description of his patent apparatus for spinal curvature, and judging from its mechanism it is a distinct advance on the unwieldy combinations of screws, ratchets and plates that are now fast becoming obsolete. The principles on which it is constructed, the support being obtained by a zig-zag steel spring in the lumbar region, commends it to modern orthopædic surgeons, who, while mainly relying on correction of faulty positions and properly regulated free exercises, still in the earlier stages of treatment require some support in the intervals between these kinetics that shall not interfere entirely with the free play of the muscles. Any distinct expression of opinion, however, as to its real practical value must be reserved until it has undergone a more extended trial; we would commend its further investigation to the members of the new British Orthopædic Society.

